# Selonsertib Alleviates the Progression of Rat Osteoarthritis: An *in vitro* and *in vivo* Study

**DOI:** 10.3389/fphar.2021.687033

**Published:** 2021-07-12

**Authors:** Jiyuan Yan, Yingchi Zhang, Gaohong Sheng, Bowei Ni, Yifan Xiao, Shanxi Wang, Tianqi Wang, Yongzhuang Ma, Huaixi Wang, Hua Wu, Chang Tu

**Affiliations:** ^1^ Department of Orthopedics, Renmin Hospital of Wuhan University, Wuhan, China; ^2^ Department of Orthopedics, Tongji Hospital, Tongji Medical College, Huazhong University of Science and Technology, Wuhan, China; ^3^ Department of Traumatology, Tongji Hospital, Tongji Medical College, Huazhong University of Science and Technology, Wuhan, China; ^4^ Department of Pathology and Pathophysiology, Medical College, Jianghan University, Wuhan, China; ^5^ Department of Orthopedics, Shanxi Bethune Hospital, Taiyuan, China; ^6^ Department of Spine and Spinal Cord Surgery, Henan Provincial People’s Hospital, People’s Hospital of Zhengzhou University, Zhengzhou, China

**Keywords:** selonsertib, osteoarthritis, apotosis, ASK1, p38, JNK, NFκB

## Abstract

Osteoarthritis (OA) is a prevalent degenerative joint disease. Its development is highly associated with inflammatory response and apoptosis in chondrocytes. Selonsertib (Ser), the inhibitor of Apoptosis Signal-regulated kinase-1 (ASK1), has exhibited multiple therapeutic effects in several diseases. However, the exact role of Ser in OA remains unclear. Herein, we investigated the anti-arthritic effects as well as the potential mechanism of Ser on rat OA. Our results showed that Ser could markedly prevent the IL-1β-induced inflammatory reaction, cartilage degradation and cell apoptosis in rat chondrocytes. Meanwhile, the ASK1/P38/JNK and NFκB pathways were involved in the protective roles of Ser. Furthermore, intra-articular injection of Ser could significantly alleviate the surgery induced cartilage damage in rat OA model. In conclusion, our work provided insights into the therapeutic potential of Ser in OA, indicating that Ser might serve as a new avenue in OA treatment.

## Introduction

Osteoarthritis (OA), featured with joint pain, stiffness and dysfunction, is the most frequently seen degenerative arthritis in the elderly (Glyn-Jones et al., 2015). Over the past decades, the prevalence of OA continues to rise globally. It is the leading cause of chronic pain and impaired physical mobility in aged population (Losina et al., 2013; Saberi Hosnijeh et al., 2019). Until now, most therapeutic strategies targeting OA work on the symptoms relief rather than disease progression reversal. Therefore, joint replacement surgery is the only choice for patients in end-stage OA (Bellamy et al., 2006). This undoubtedly brings huge burden to the society and individuals.

Despite OA is a complex process which pathogenesis involves aging, mechanical injury, inflammatory response and metabolic dysfunction, inflammation plays a critical role in OA course (Hunter and Bierma-Zeinstra, 2019). Reportedly, local inflammatory stimulation and cartilage metabolism disorders contribute to the progression of OA (de Lange-Brokaar et al., 2012; van den Bosch, 2021). Accumulating evidence has showed that excessive pro-inflammatory cytokine, especially interleukin-1β (IL-1β) was detected in the synovial fluid from OA patients (Kellesarian et al., 2016). Previous study confirmed that IL-1β treatment could increase the expression of metallo-proteinases (MMPs) and aggrecanase-2 (ADAMTS5) in chondrocytes, thus resulting in the cartilage matrix damage (Tu et al., 2019b). Meanwhile, elevated dose of IL-1β could trigger the severer inflammatory reaction due to overproduction of inflammatory mediators such as inducible nitric oxide synthase (iNOS) and cyclooxygenase2 (COX2) (Tu et al., 2019a). Programmed cell death apoptosis plays important roles in maintaining cartilage homeostasis (Carames et al., 2015). Excessive apoptosis due to local inflammatory microenvironment is a great challenge in OA therapy (Dai et al., 2018). It is proved that IL-1β could markedly induce the mitochondrial dysfunction-related apoptosis in chondrocytes (Wang et al., 2020). Moreover, oral gavage of apoptosis inhibitors administration could effectively alleviate the cartilage damage in mice OA model (Wang et al., 2019). Therefore, regents against IL-1β may provide breakthrough in OA therapy.

Selonsertib (Ser), a selective inhibitor of Apoptosis Signal-regulated kinase-1 (ASK1), has earned its reputation for its anti-inflammatory and anti-apoptotic properties in nonalcoholic steatohepatitis therapy (Loomba et al., 2018). In addition, it is reported that Ser could effectively slow diabetic kidney disease progression (Chertow et al., 2019; Cuarental et al., 2019). However, the detailed role of Ser in OA treatment remains to explore. Previous study reported that Ser could block ASK1/MAPK pathway in hepatic stellate cells and down-regulate ASK1-JNK-DRP1 pathway in macrophage, suggesting the possible mechanism of its action (Yoon et al., 2020; Lou et al., 2021). In this study, we aim to elucidate the therapeutic effects and molecular mechanisms of Ser in OA. We expect to excavate promising strategies in OA treatment.

## Materials and Methods

### Ethics Approval

This study was performed in strict accordance with the Guidelines of Animal Care and Use Committee for Teaching and Research, Tongji Medical College, Huazhong University of Science and Technology. All experimental protocols were confirmed by the Institutional Animal Care and Use Committee, Tongji Medical College, Huazhong University of Science and Technology. All efforts were conducted to minimize animal suffering.

### Regents

Selonsertib (purity: 99.4%) was purchased from Selleck Chemicals (United States). DMSO was used to dissolve selonsertib, and equal volume of DMSO was added to all experiment groups. Dulbecco’s modified Eagle’s medium F12 (DMEM/F12) and Fetal bovine serum (FBS) were procured from Gibco (NY, United States). Recombinant rat IL-1β was acquired from R&D systems (MN, United States). Antibodies specific for P-P65 (#3003), COX2 (#12882), P-ASK1 (#3764), ASK1 (#8662), P-P38 (#4511), P38 (#8690), P-JNK (#9255), JNK (#9258), P-IκBα (#5209) were purchased from Cell Signaling Technology (MA, United States). Antibodies against C-caspase3 (#PB0183) MMP3 (#BM4074), ADAMTS5 (#BA3020), GAPDH (#BM3876), β-ACTIN (#BM0627) were obtained from Boster (Wuhan, China). Antibodies specific for P65 (#10745-1-AP), P53 (#60283-2-Ig), Collagen Ⅱ (#15943-1-AP), Bcl-XL (#10783-1-AP), BAX (#50599-2-Ig) were purchased from Proteintech Group (Wuhan, China). Antibody against C-caspase 9 (#A0281) was procured from ABclonal (Wuhan, China). Antibody against MMP13 (ab84594) was obtained from Abcam (MA, United States). Antibody against iNOS (#sc-7271) was purchased from Santa Cruz Biotechnology (CA, United States).

### Chondrocytes Isolation and Culture

Primary rat chondrocytes were isolated from 1-week old Sprague-Dawley (SD) male rat as described before (Ma et al., 2019). Concisely, cartilage was obtained from the bilateral knee joints and minced into small pieces. Then the pieces were firstly digested with 0.25% trypsin for 30 min at room temperature. Subsequently, 0.25% collagenase Ⅱ was added to fully digest the cartilage fragments overnight. Afterwards, the cell suspension was collected and centrifuged at a speed of 1,500 rpm. The precipitated cell pallet was taken after centrifugation. Finally, cells were cultured in DMEM/F12 containing 10% FBS and 1% penicillin/streptomycin solution. Chondrocytes at passage two or three were used in subsequent experiment.

### Cell Viability

Effects of Ser on rat chondrocyte viability were detected with a Cell Counting Kit-8 (CCK-8) assay. Briefly, cells were seeded into 96-well plates at a density of 10,000 cells/well. After adhesion, cells were treated with various doses of Ser (0, 5, 10, 25, 50 μM) alone or in combination with IL-1β (10 ng/ml) for 24 h. Then, cells in each well were treated with 10 μL CCK-8 solutions and incubated for 1 h in dark. After incubation, the absorbance of each well was measured at 450 nm using a microplate reader (Bio-Rad, CA, United States).

### Western Blotting

Chodrocytes were seeded onto 6-well plates at a density of 2 × 10^5^ cells/well for further research. Cells were washed with phosphate buffered saline (PBS) twice and lysed in RIPA solutions supplemented with 1% protease/phosphatase inhibitor cocktail. Then, a bicinchoninic acid (BCA) kit was used to determine the protein concentration. Subsequently, each amount of protein samples (30 μg) were separated on 8–12% SDS-PAGE gels, transferred onto PVDF membranes and blocked in 5% BSA for 1 h at 37°C. Afterwards, the membranes were incubated with the corresponding primary antibodies at 4°C overnight. After washing with TBST, the membranes were next incubated with indicated secondary antibodies for 1 h at room temperature. Finally, the bounded protein was visualized using enhanced ECL kit. All bands were photographed using a Bio-Rad scanner system and quantified with the Image-J software.

### Measurement of Cell Apoptosis

Cells were seeded onto 6-well plates at a density of 2 × 10^5^ cells/well for futher research. An Annexin V-FITC/PI Apoptosis Detection kit (Beyotime, China) was introduced to measure the chondrocytes apoptosis. According to the manufacturer’s instruction, rat chondrocytes were collected and washed with cold PBS twice. Then, cells were resuspended in binding buffer and transferred to centrifuge tubes. Subsequently, cells were stained with Annexin V-FITC/PI for 15 min in dark. The apoptotic chondrocytes were analyzed with a FACScan flow cytometer (United States). The sum of Annexin V+/PI+ and Annexin V+/PI- chondrocytes were considered as apoptotic cells.

### Immunofluorescence

Chondrocytes were seeded into 24 well plates for P65 immunofluorescence staining at a density of 10,000 cells/well. After reaching 50% confluence, cells were treated with 10 ng/ml IL-1β alone or in combination of 25 μM Ser. Then, cells were fixed with 4% paraformaldehyde for 15 min and washed with PBS three times. Subsequently, cells were permeabilized by 0.2% Triton X-100 for 10 min and blocked by 5% BSA for 1 h. Next, the chondrocytes were incubated with antibody against P65 at 4°C overnight. Afterwards, cells were washed with PBS and incubated with Cy3-conjugated secondary antibody for 1 h at room temperature in dark. Finally, cells were mounted with DAPI for 10 min and observed using a fluoresecence microscope (Evos FL auto, United States).

### Animal Osteoarthritis Model

Eighteen male 2-month-old SD rats were supplied from the Laboratory Animal Center of Tongji Hospital. Anterior cruciate ligament transection (ACL-T) and partial medial meniscus removal was performed on the right knee to built OA model as reported previously (Chu et al., 2013). All animals were randomly divided into sham, Ser and OA groups. For sham group (*n* = 6), rats accepted sham operation without ACL-T or medical meniscus removal. For Ser group (*n* = 6), rats accepted surgery and intra-articular injection of 25 μM Ser once a week. For OA group (*n* = 6). Rats accepted surgery and intra-articular injection of equal volume of sterile saline once a week. All animals were sacrificed at 2 months post surgery. The knee joints samples were fixed in 4% paraformaldehyde for next analysis.

### Histological Assessment

Fixed joints were decalcidied using 10% EDTA solution for 2 weeks, embedded in paraffin and cut into 5 μm thickness sections coronally. Then sections were stained with Safranin-O-Fast green and H&E. The Osteoarthritis Research Society International (OARST) grading system was introduced to assess the OA changes in a blinded manner. Immunohistological staining was further conduted using antibodies specific for COX2, MMP13, Collagen Ⅱ, and Cleaved-caspase 3.

### Statistical Analysis

Values are expressed as mean ± standard deviation (SD). All of the data analysis were performed using one-way analysis of variance (ANOVA) followed by a Turkey’s post hoc test. *p*-values ˂ 0.05 were considered statistically significant. All *in vitro* experiments were conducted at least in triplicate.

## Results

### Effects of Ser on Cell Viability

**FIGURE 1:**
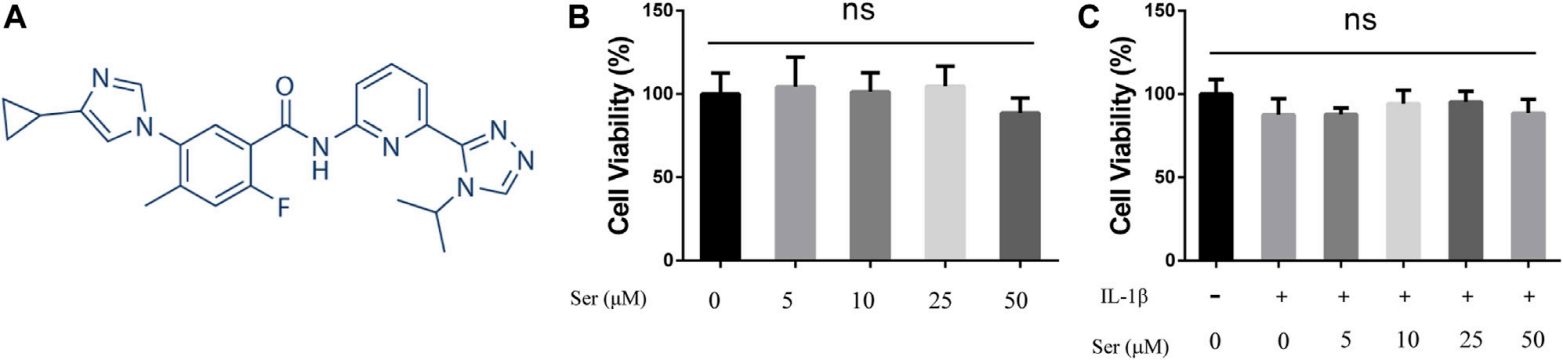
Effects of Ser on Cell Viability. **(A)** Chemical structure of Ser. **(B)** Rat chondrocytes were treated with different concentrations of Ser in the absence or **(C)** presence of IL-1β (10 ng/ml) for 24 h. Cell viability was evaluated using CCK-8 assay. NS indicated no significance.

The cytotoxic effects of different doses of Ser with or without IL-1β (10 ng/ml) on cultured rat chondrocytes were determined via CCK-8 assay. As shown in Figure 1, Ser (concentrations of 5, 10, 25, 50 μM) alone or Ser (concentrations of 5, 10, 25 μM) combined with 10 ng/ml IL-1β had no toxic effects on cultured cells. Ser at the dose of 25 μM was used in subsequent study.

### Ser Attenuates IL-1β-Induced Inflammatory Responses and Cartilage Matrix Degradation in Chondrocytes

**FIGURE 2:**
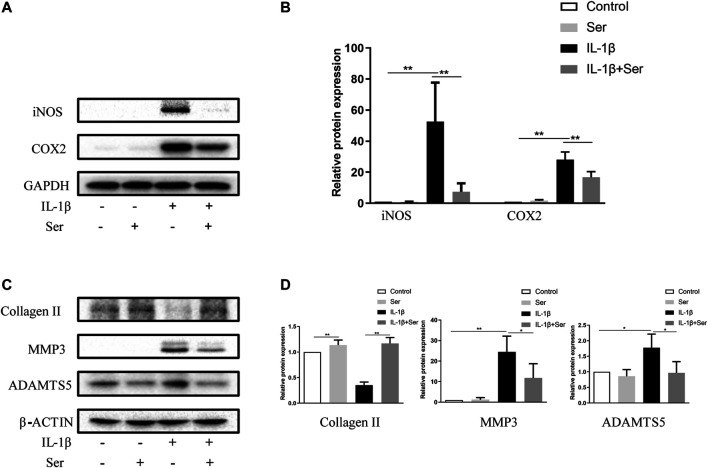
Ser attenuates IL-1β-induced inflammatory responses and cartilage matrix degradation in chondrocytes. Chondrocytes were treated with Ser (25 μM) in the absence or presence of IL-1β (10 ng/ml) for 24 h. **(A)** Western blot and **(B)** quantitative analysis of iNOS and COX2. **(C)** Western blot and **(D)** quantitative analysis of collagen Ⅱ, MMP3 and ADAMTS5. GAPDH and β-ACTIN were used as the external control respectively. ^∗^ *p* < 0.05,^∗∗^ *p* < 0.01, *n* = 3.

To investigate whether Ser could ameliorate inflammatory responses and cartilage matrix degradation induced by IL-1β, western blotting was conducted. As shown in Figures 2A,B, administration of 25 μM Ser could notably attenuate the overexpression of INOS and COX2 induced by IL-1β treatment in cultured chondrocytes. Moreover, as shown in Figures 2C,D, administration of IL-1β significantly induce the upregulation of MMP13, ADMTS5 and the downregulation of collagen Ⅱ. However, 25 μM Ser treatment could partly reverse this change.

### Ser Ameliorates IL-1β-Induced Apoptosis in Chondrocyte

**FIGURE 3:**
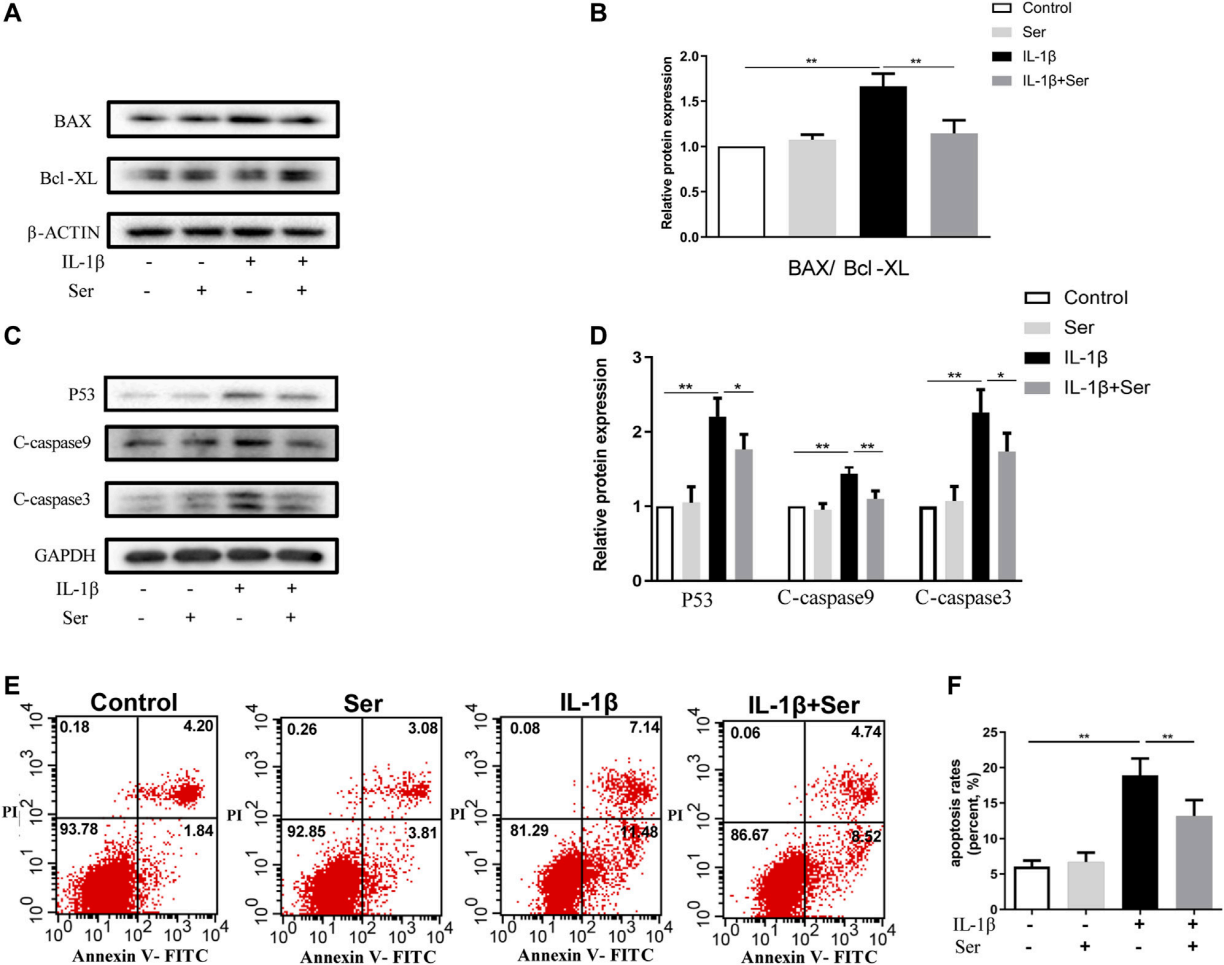
Ser ameliorates IL-1β-induced apoptosis in rat chondrocytes. Cells were treated with Ser (25 μM) alone or in combination with IL-1β (10 ng/ml) for 24 h. **(A)** Western blot of the expression of BAX, Bcl-XL **(B)** quantitative analysis BAX/Bcl-XL. **(C)** Western blot and **(D)** quantitative analysis of P53, C-caspase9 and C-caspase3. GAPDH was used as the loading control. **(E)** Apoptotic cells were detected with Annexin V-FITC/PI staining and flow cytometry. **(F)** Quantitative of the apoptosis rates of each group. ^∗^ *p* < 0.05, ^∗∗^ *p* < 0.01, *n* = 3.

Apoptosis acts as an important role in maintaining cartilage homeostasis. To observe the effects of Ser on cultured chondrocyte apoptosis induced by IL-1β, we first detected apoptosis related proteins using western blotting. As shown in Figures 3A,B, Ser (25 μM) could effectively reverse the elevated ratio of BAX/Bcl-XL induced by IL-1β. Besides, 25 μM Ser could attenuate the IL-1β-induced upregulation of P53, C-caspase9 and C-caspase3 (Figures 3C,D). The Annexin V-FITC/PI Apoptosis Detection kit was performed to measure apoptosis levels in cultured chondrocytes. As exhibited in Figures 3E,F, administration of Ser caused a notable decrease of apoptotic cells induced by IL-1β.

### Effects of Ser on IL-1β-Induced ASK1/P38/JNK Pathway Activation in Chondrocytes

**FIGURE 4:**
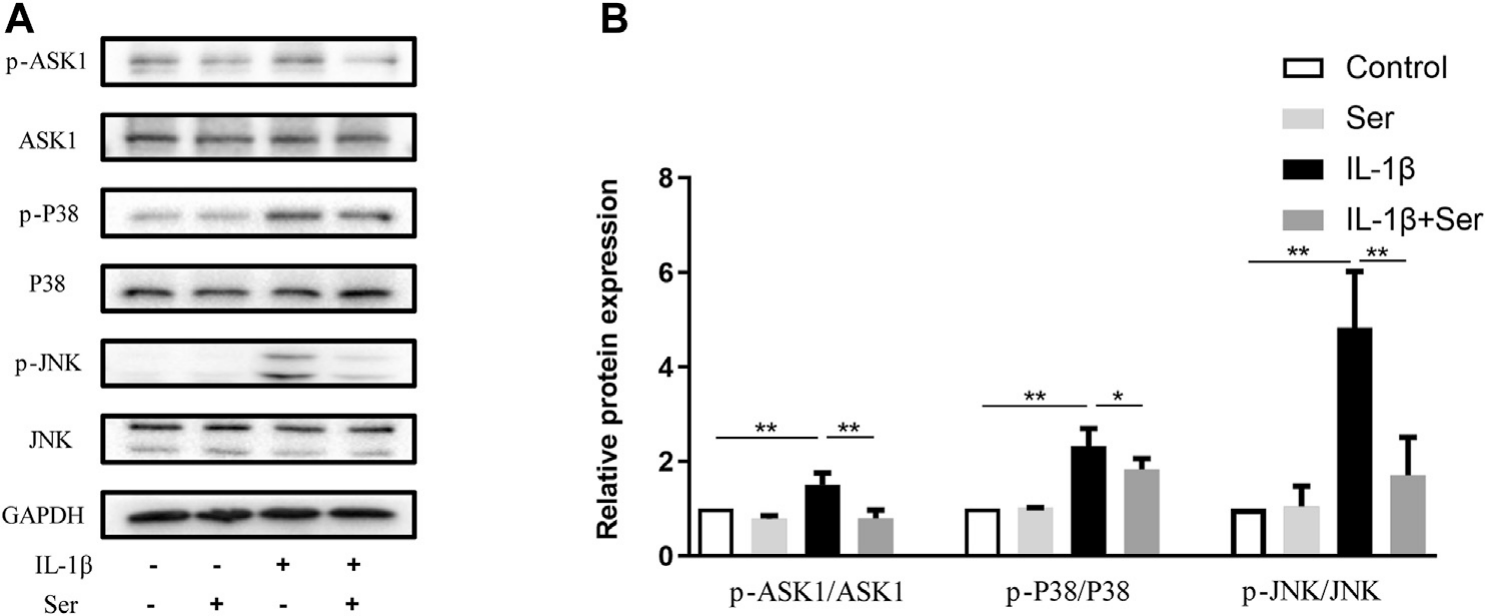
Ser blocks the IL-1β-induced ASK1/P38/JNK activation in chondrocyte. Cells were exposed to L-1β (10 ng/ml) with or without 25 μM Ser for 30 min. **(A)** Western blot and **(B)** quantitative analysis of ASK1/P38/JNK pathway in each group as above. ASK1, P38 and JNK were used as the external control. ^∗^ *p* < 0.05, ^∗∗^ *p* < 0.01, *n* = 3.

ASK1/P38/JNK pathways play important roles in modulating cell proliferation and inflammation response. In present study, we investigated the exact role of Ser on IL-1β-induced ASK1/P38/JNK pathway activation in cultured chondrocytes. As shown in Figure 4 A, B, 25 μM Ser could significantly block the phosphorylation of ASK1/P38/JNK in IL-1β-treated rat chondrocytes.

### Effects of Ser on IL-1β-Induced NFκB Pathway Activation in Chondrocytes

**FIGURE 5:**
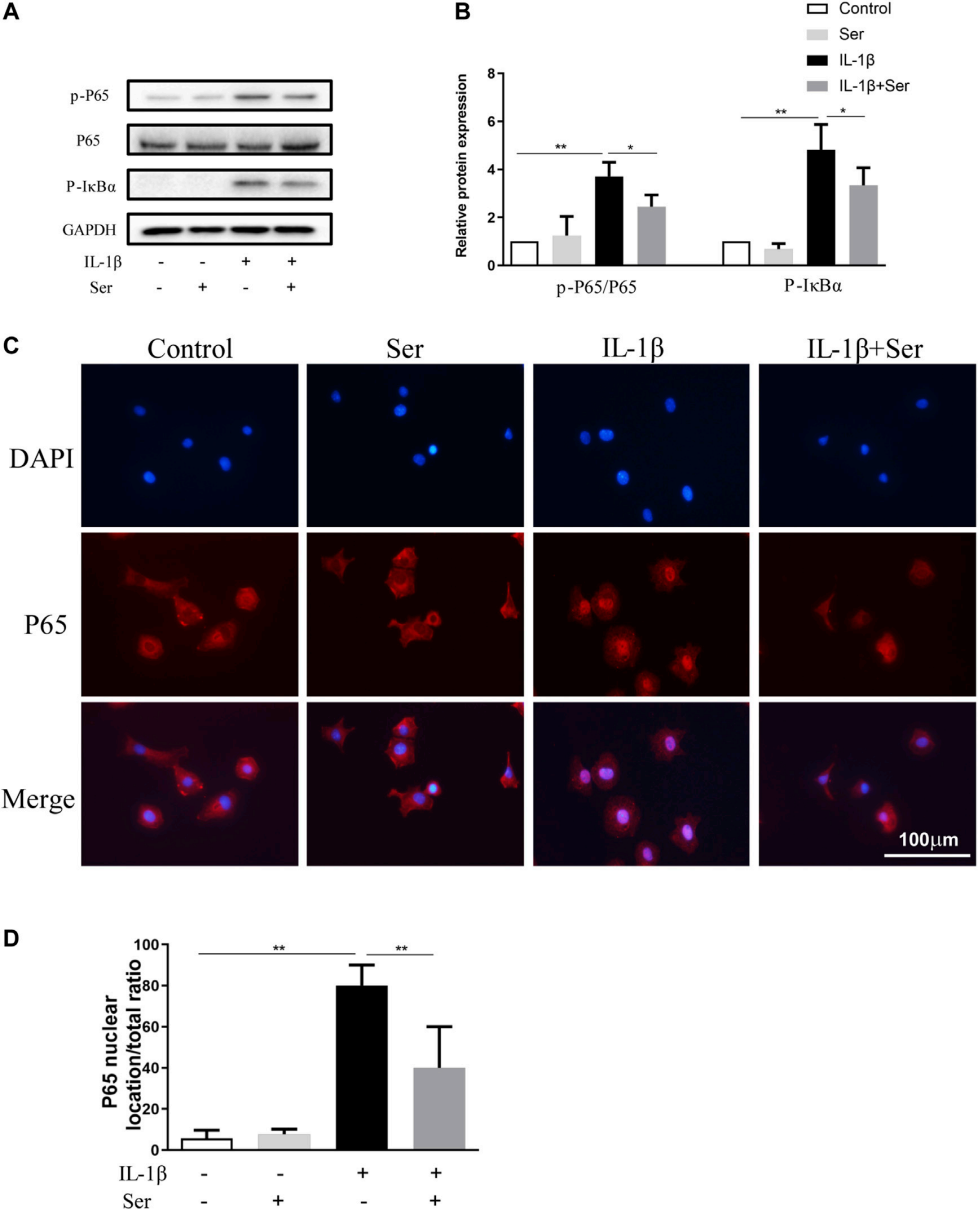
Ser blocks the activation of NFκB pathway in IL-1β-treated chondrocyte. Cells were exposed to L-1β (10 ng/ml) with or without 25 μM Ser for 30 min. **(A)** Western blot and **(B)** quantitative analysis of P-P65 and P-IκBα. P65 and GAPDH were used as the loading control respectively. **(C)** P65 nuclear translocation was detected using immunofluorescence. **(D)** Quantitative analysis of P65 nuclear location ratio of each group. ^∗^ *p* < 0.05, ^∗∗^ *p* < 0.01, *n* = 3.

NFκB pathway is highly involved in the progression of OA. In present study, western blotting was performed to detect the activation of NFκB P65, IκBα and immunofluorescence staining was employed to evaluate P65 nuclear translocation. As shown in Figures 5A,B, IL-1β (10 ng/ml) could dramatically induce the phosphorylation of P65 and IκBα in cultured chondrocytes, while Ser could partly reverse this change. Furthermore, 25 μM Ser could notably inhibit the IL-1β-induced P65 nuclear translocation in cultured rat chondrocytes (Figures 5C,D).

### Ser Prevents Cartilage Damage in Rat Osteoarthritis Model

**FIGURE 6:**
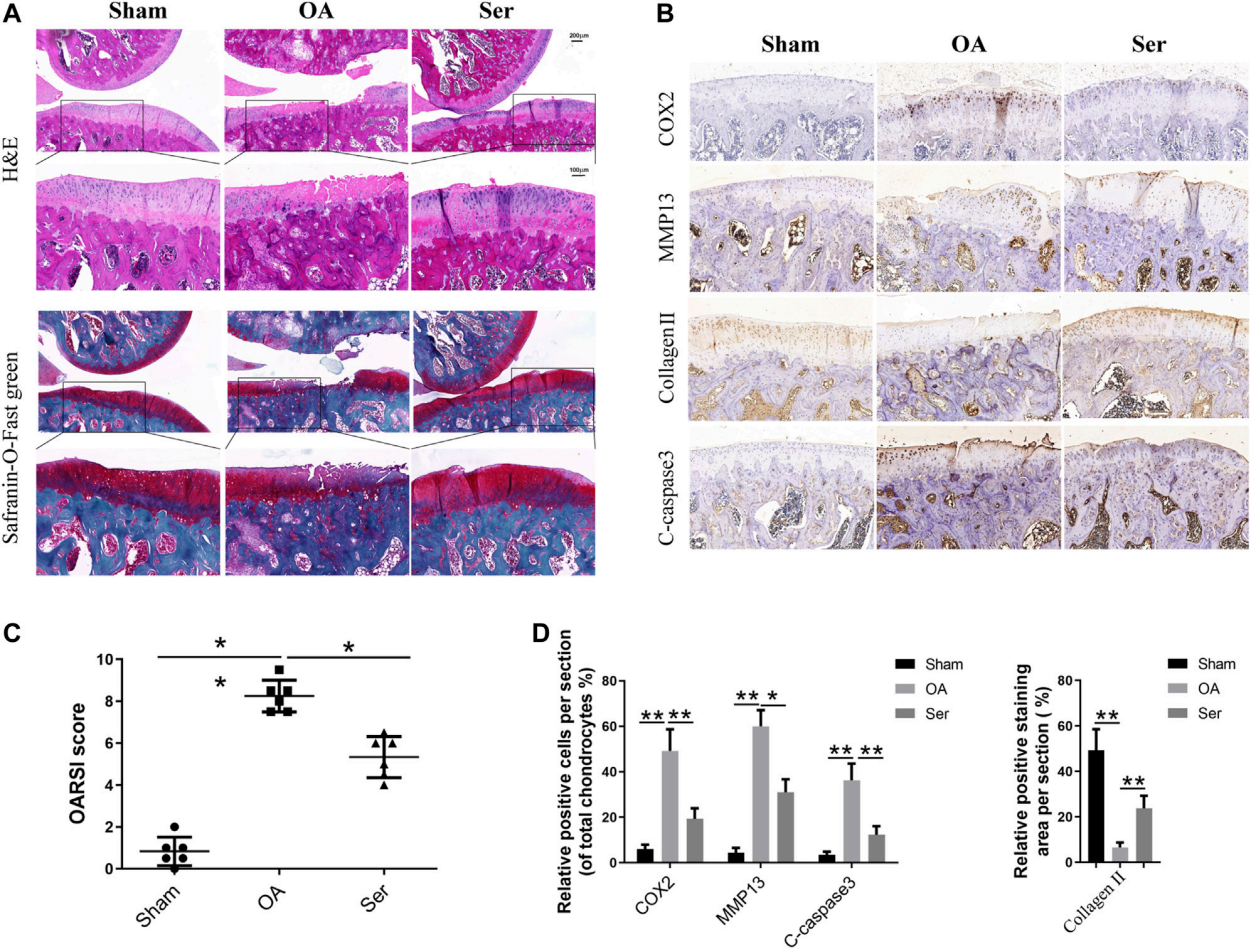
Ser prevents cartilage damage in rat OA model. **(A)** H&E and Safranin-O-Fast green staining of cartilage samples from each group 8 weeks post sugery. **(B)** OARIS scores of each group. **(C)** Immunohistochemical staining and **(D)** quantitative of COX2, MMP13, collagen Ⅱ and C-caspase3 in the cartilage samples from each group. ^∗^ *p* < 0.05, ^∗∗^ *p* < 0.01.

To manifest the effects of Ser on the pathogenesis of rat OA *in vivo*, we established rat OA model via anterior cruciate ligament transection and partial medial meniscus removal. As shown in Figure 6A, extensive cartilage erosions, proteoglycan loss and chondrocytes disorganization were observed in OA group, while cartilage in Ser group exhibited a smoother and more intact structure. Moreover, a lower OARSI score was seen in Ser group compared to OA group (Figure 6B). The immunohistochemistry staining analysis indicated the consistent trend with the vitro study (Figures 6C,D). Intra-articular injection of 25 μM Ser could markedly attenuate inflammatory reaction, cartilage matrix degradation and chondrocyte apoptosis *in vivo*.

## Discussion

Osteoarthritis (OA) is a degenerative joint disorder with high incidence in the elderly. Due to shortage of effective treatments for revsersing the progression of OA, symptomatic relief such as killing pain is routinely provided for early stage patient (Mandl, 2019). Recently, selonsertib (Ser), a selective inhibitor of Apoptosis Signal-regulated kinase-1 (ASK1), has attracted wide close attention for its anti-apoptosis and anti-inflammation effects in nonalcoholic steatohepatitis (NASH) treatment (Younossi et al., 2018). In present study, we firstly report the therapeutic potential and related molecular mechanisms of Ser in rat OA.

IL-1β is a key pro-inflammatroy cytokine involved in the pathogenesis of OA (Kapoor et al., 2011). Massive IL-1β is secreted in the joint of OA patients (Kellesarian et al., 2016). Large amount of evidence suggested that IL-1β could induce severe inflammatory reaction and cartilage matrix degradation in OA (Tu et al., 2019a; Jenei-Lanzl et al., 2019). Moreover, *in vitro*, IL-1β exhibited the strongest reaction effects in rat chondrocytes at a dose of 10 ng/ml (Wang et al., 2018). Therefore, IL-1β treatment (10 ng/ml) was introduced as a stimulus in our vitro study. Our data revealed that Ser could antagonize IL-1β induced production of inflammatory mediators including INOS and COX2. Besides, the IL-1β induced downregulation of collagen Ⅱ and upregulation of MMP13 and ADAMTS5 were also blocked by Ser. Summarily, we have proved the anti-inflammatory as well as the anti-degenerative effects of Ser on IL-1β-treated rat chodrocytes.

Apoptosis plays a crucial role in pathogenesis of OA. Elevated level of apoptosis results in the compromising of chondrocyte surval and function (Hwang and Kim, 2015). Previous study confirmed that apoptosis was promoted by caspases, which was regulated by Bcl-2 family (Boise et al., 1995). In the Bcl-2 family, Bcl-XL represents the anti-apoptotic subfamily while BAX represents the pro-apoptotic one (Elmore, 2007). Activated P53 transcriptionally promotes the apoptosis, and acts as a key regulator in this process (Hafner et al., 2019). In this study, we showed that Ser could effectively reduce the amount of apoptotic chondrocytes induced by IL-1β. Moreover, Ser had been shown to alleviate the IL-1β-induced increase of P53, C-caspase9, C-caspase3 and BAX/Bcl-XL ratio. Taken together, the vitro anti-apoptotic effects of Ser on rat OA was confirmed.

Attempts to prevent P38 or JNK mediated disorders using specific inhibitors of P38 or JNK have been proved unsuccessful. ASK1, the upstream regulator of P38 and JNK, has been proposed as the promising drug target in multiple inflammatory diseases including OA (Ogier et al., 2020). As a selective inhibitor of ASK1, Ser might work via regulating ASK1/P38/JNK pathways in OA. Besides, activation of NFκB is ubiquitous in OA progression. Proinflmmatory cytokines like IL-1β could trigger the phosphorylation of IκBα, thus contributing to the production of inflammatory factors and metallo-proteinases (MMPs) in OA (Baldwin, 2001; Roman-Blas and Jimenez, 2006). Therefore, in our study, we focused on these two pathways. Our work showed Ser could significantly block the activation of ASK1/P38/JNK and NFκB signal pathways induced by IL-1β in rat chondrocytes. The observations partly mimic results of Yoon et al. study, which demonstrated that selonsertib alleviates liver fibrosis via blocking ASK1/MAPK pathway in hepatic stellate cells (Yoon et al., 2020). Collectively, the two pathways were involved in the protective roles of Ser in OA. To illustrate the therapeutic potential of Ser in OA, vitro testing is far from enough. We further built rat OA model and assessed the protective effects of Ser *in vivo*. Data proved intra-articular injection of Ser could effectively alleviate the progression of rat OA.

Some limitations should be pointed out. OA is a complex disease caused by mutilple pathological factors (Hunter and Bierma-Zeinstra, 2019). We mainly focused on the role of Ser on the inflammation during OA in this study, and the effects of Ser on other pathological changes in OA remains further investigation. Besides, it has been reported that Ser failed in the phase III stellar clinical trials (Harrison et al., 2020). Although the pathogenesis of OA and NASH is quite different and the protective effects of Ser on rat OA is promising, it is necessary to assess the safety and efficacy of Ser in lager animals and human OA model. Moreover, in this study, we failed to conduct the serological test and observe the subchondral bone change. Furthermore, the direct target of Ser in OA remains unknown. In view of the achievement we have completed, further exploration is needed.

## Conclusion

In conlusion, we are the first to report the anti-inflammatroy, anti-degenerative and anti-apoptotic effects of Ser in rat OA. We also revealed the related pathways involved in this process, indicating the underlying mechanisms. Our vivo study proved that Ser may serve as a promising candidate in OA therapy via delaying cartilage damage and degradation. We believe our study will provide insight into the OA therapy as well as supplement to the pharmacology of Ser.

## Data Availability

The raw data supporting the conclusions of this article will be made available by the authors, without undue reservation.
