# Supplementary material for: Selonsertib Alleviates the Progression of Rat Osteoarthritis: An in vitro and in vivo Study
Source: Front Pharmacol. 2021 Jul 12;12:687033. doi: 10.3389/fphar.2021.687033 (PMC8311523; doi:10.3389/fphar.2021.687033)
Supplement: Supplementary file 1 [file Table1.DOCX]

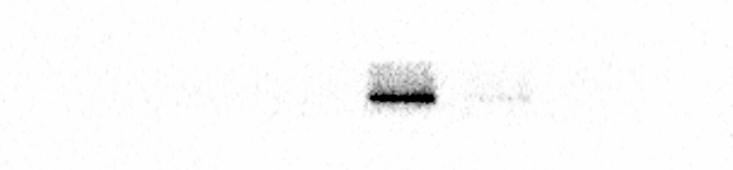


iNOS (fig.2A)


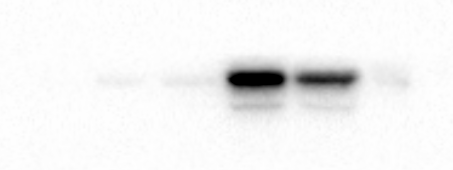


COX2 (fig.2A)


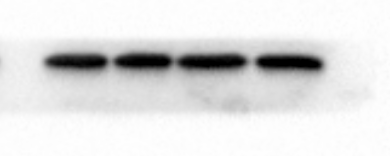


GAPDH (fig.2A)


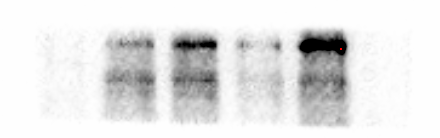


Collagen Ⅱ(fig.2C)


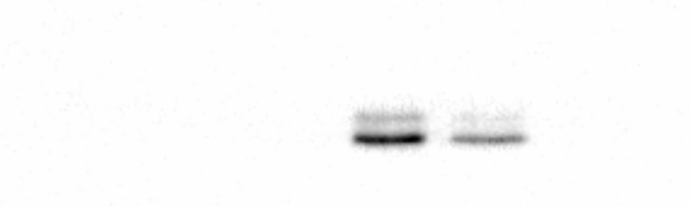


MMP3 (fig.2C)


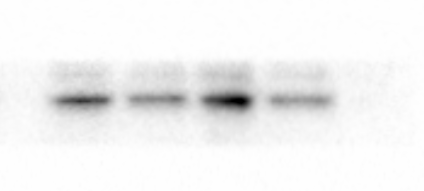


ADAMTS5 (fig.2C)


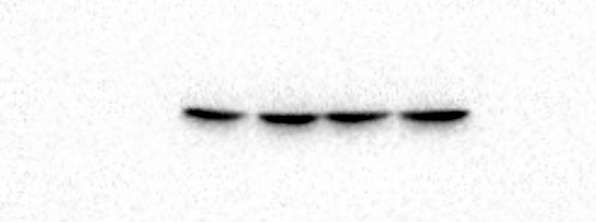


Β-ACTIN (fig.2C)


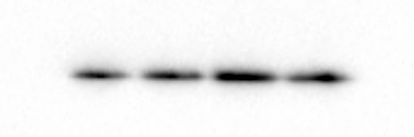


BAX (fig.3A)


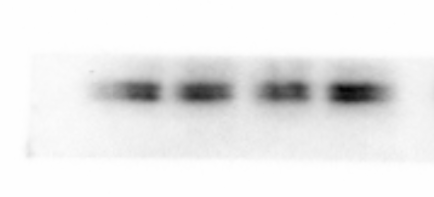


Bcl-XL (fig.3A)


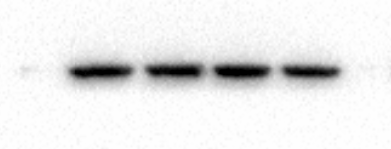


Β-ACTIN (fig.3A)


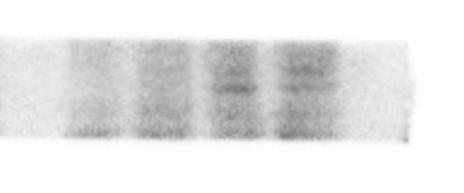


P53 (fig.3C)


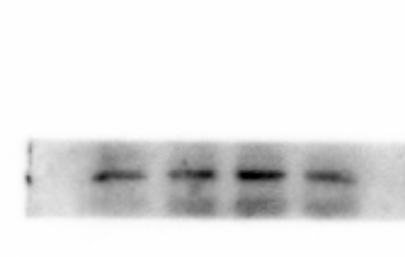


C-caspase9 (fig.3C)


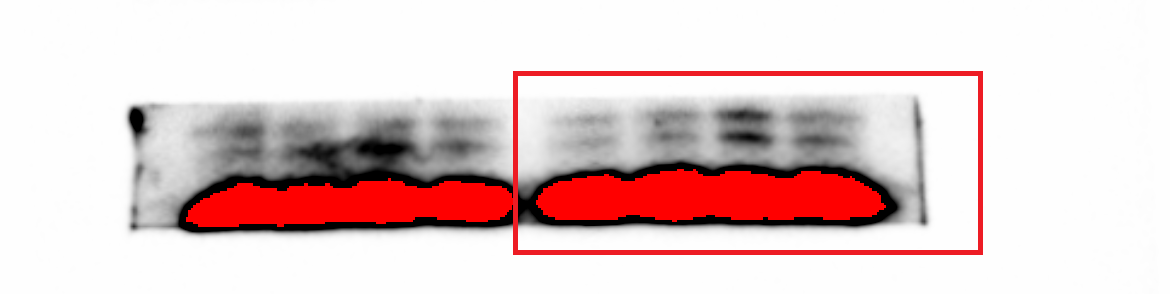


C-caspase 3 (fig.3C)


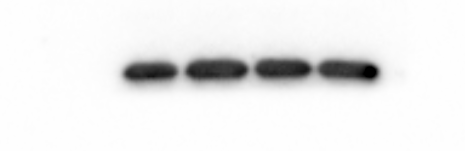


GAPDH (fig.3C)


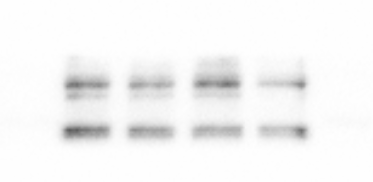


P-ASK1 (fig.4A)


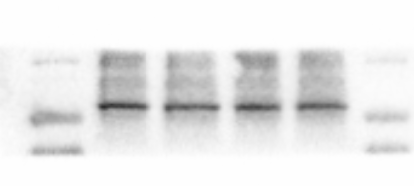


ASK1 (fig.4A)


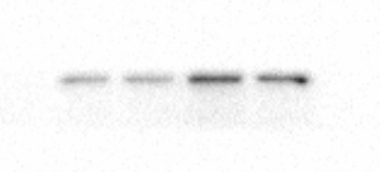


P-P38 (fig.4A)


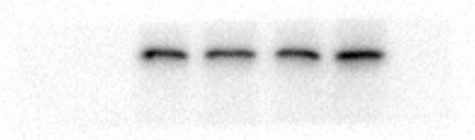


P38 (fig.4A)


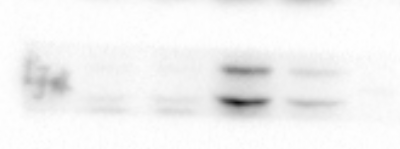


p-JNK (fig.4A)


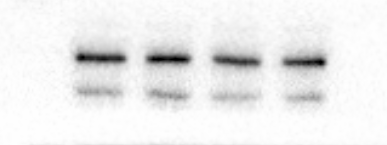


JNK (fig.4A)


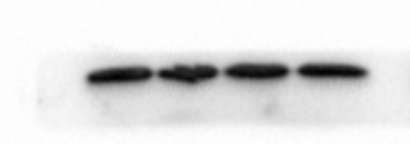


GAPDH (fig.4A)


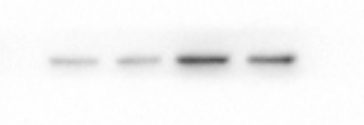


P-P65 (fig.5A)


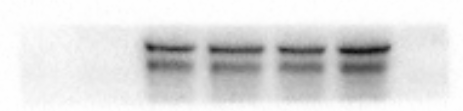


P65 (fig.5A)


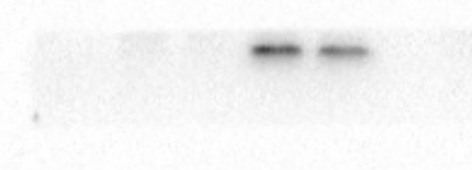


P-IκBα (fig.5A)


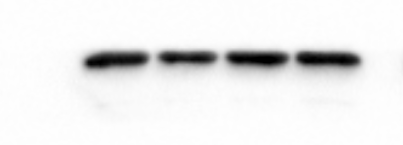


GAPDH (fig.5A)
